# Fast-running theropods tracks from the Early Cretaceous of La Rioja, Spain

**DOI:** 10.1038/s41598-021-02557-9

**Published:** 2021-12-09

**Authors:** Pablo Navarro-Lorbés, Javier Ruiz, Ignacio Díaz-Martínez, Erik Isasmendi, Patxi Sáez-Benito, Luis Viera, Xabier Pereda-Suberbiola, Angélica Torices

**Affiliations:** 1 Cátedra Extraordinaria de Paleontología, Departamento de Ciencias Humanas, Universidad de La Rioja (UR), C/Luis de Ulloa, 2, 26004 Logroño, La Rioja Spain; 2 Departamento de Geodinámica, Estratigrafía y Paleontología, Universidad Complutense de Madrid, 28040 Madrid, Spain; 3 Universidad Nacional de Río Negro-IIPG. Av, Roca 1242, 8332 General Roca, Río Negro Argentina; 4 Instituto de Investigación en Paleobiología y Geología (IIPG), CONICET. Av. Roca 1242, 8332 General Roca, Río Negro Argentina; 5 Departamento de Geología, Facultad de Ciencia y Tecnología, Universidad del País Vasco/Euskal Herriko Unibertsitatea (UPV/EHU), Barrio Sarriena S/N., 48940 Leioa, Bizkaia Spain; 6 Centro de Interpretación Paleontológica de la Rioja, Calle Mayor, 10, 26525 Igea, La Rioja Spain

**Keywords:** Palaeontology, Biological physics

## Abstract

Theropod behaviour and biodynamics are intriguing questions that paleontology has been trying to resolve for a long time. The lack of extant groups with similar bipedalism has made it hard to answer some of the questions on the matter, yet theoretical biomechanical models have shed some light on the question of how fast theropods could run and what kind of movement they showed. The study of dinosaur tracks can help answer some of these questions due to the very nature of tracks as a product of the interaction of these animals with the environment. Two trackways belonging to fast-running theropods from the Lower Cretaceous Enciso Group of Igea (La Rioja) are presented here and compared with other fast-running theropod trackways published to date. The Lower Cretaceous Iberian fossil record and some features present in these footprints and trackways suggest a basal tetanuran, probably a carcharodontosaurid or spinosaurid, as a plausible trackmaker. Speed analysis shows that these trackways, with speed ranges of 6.5–10.3 and 8.8–12.4 ms^−1^, testify to some of the top speeds ever calculated for theropod tracks, shedding light on the question of dinosaur biodynamics and how these animals moved.

## Introduction

One of the perennial questions in the paleobiology of non-avian theropod dinosaurs is their capacity for locomotion, e.g.^1,2^. How did they move? How fast did they go? Over the years, these questions have been approached from various points of view based on osteological information, with anatomical (e.g., morphology, muscular attachments, size) and anatomically-derived biomechanical models (e.g., mass, force, and momentum) being used to estimate the maximum speed of locomotion^3–7^. Another way of better understanding how extinct theropods moved is to examine their tracks and trackways, e.g.^8^. To this end, Alexander^9^ proposed an equation using dynamic similarity to calculate the absolute speed of dinosaurs from ichnological data on the basis of footprint length (to obtain the height at the hip) and stride length. This and other methods e.g.^10,11^ have been used in the last few decades by many ichnologists to analyse the locomotion dynamics shown by hundreds of trackways, e.g.^11–14^.

**Figure 1:**
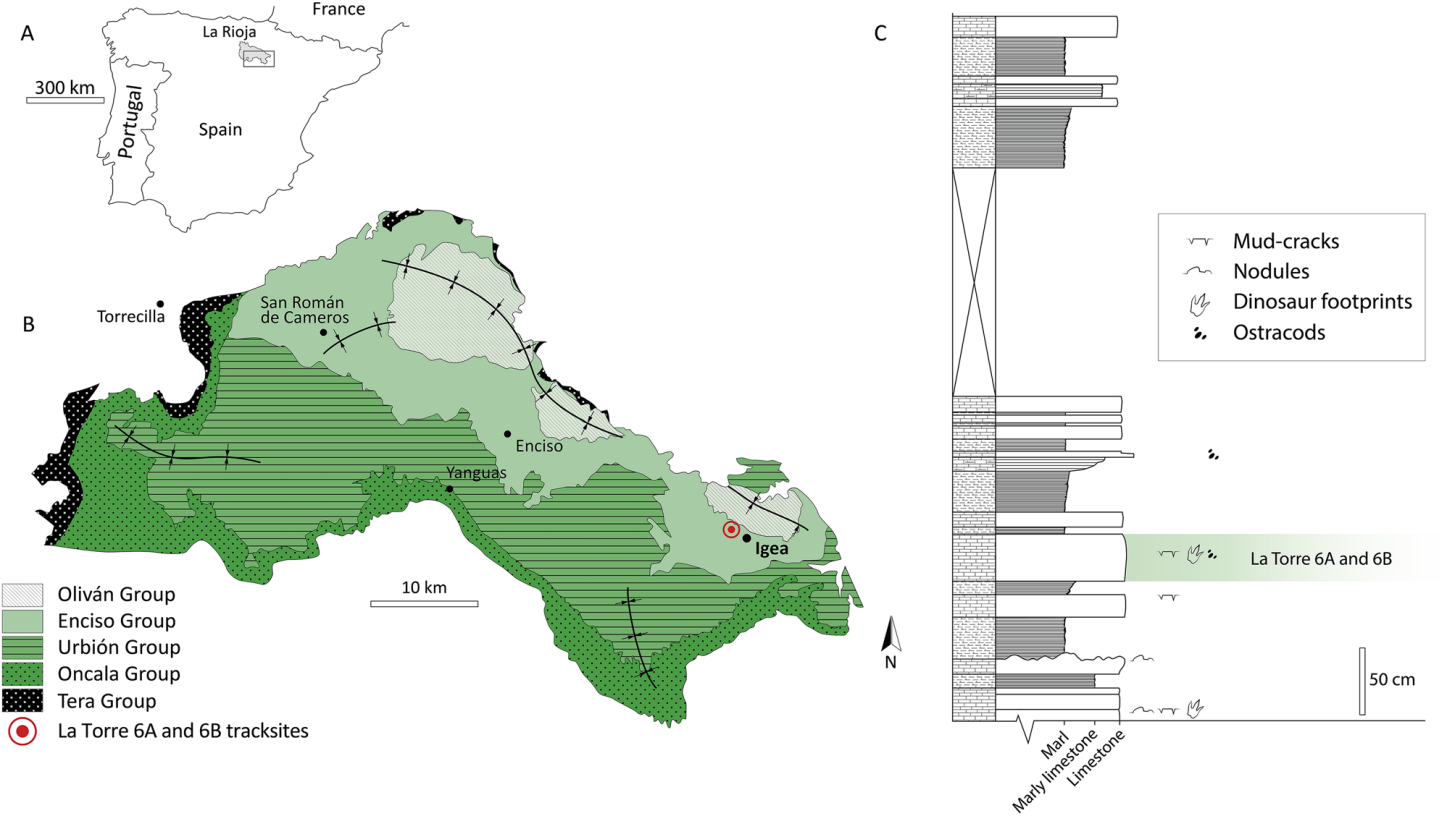
Geographical and geological location of La Torre 6A and 6B tracksites. (**A**) Location of La Rioja in the Iberian Peninsula. (**B**) Geological map of the southern part of La Rioja, with the main stratigraphical groups differentiated. (**C**) Local stratigraphic succession of the study area (modified from Isasmendi et al.^28^).

Walking is the most common behaviour inferred from dinosaur fossil trackways^9–11,15^, although some minor cases of running or trotting have also been identified^13,16–23^. Indeed, 96% of the 1802 *Kayentapus* dinosaur strides studied by Weems ^13^ were made by animals with a walking behaviour, whereas just 4% of them were made by dinosaurs with a more energetic way of movement. Of this 4%, the great majority is consistent with trotting displacement, whereas just one of the trackways could correspond to a running behaviour^13^. In the Early Cretaceous of Spain, a theropod trackway of six consecutive footprints with pace lengths of more than two metres preserved in a trampled surface was found at La Torre 6B (Igea, La Rioja)^24^ (Fig. 1), for which has been inferred high speeds of more than 10 ms^−1^ (Refs.^25–27^). The trackway from La Torre 6A was initially mentioned by Aguirrezabala et al.^24^ with the presence of two non-consecutive footprints and the probable presence of a third between them, lost by erosion. During new field campaigns in this area, two significant findings have recently been made: a new footprint was discovered to add to the La Torre 6B trackway, and the discovery of three new footprints in La Torre 6A that confirm the presence of a second high-speed trackway in La Torre tracksites. Both trackways shed light on locomotion, speeds and even behaviour of non-avian theropods.

## Results and discussion

### Tracks and trackways

**Figure 2:**
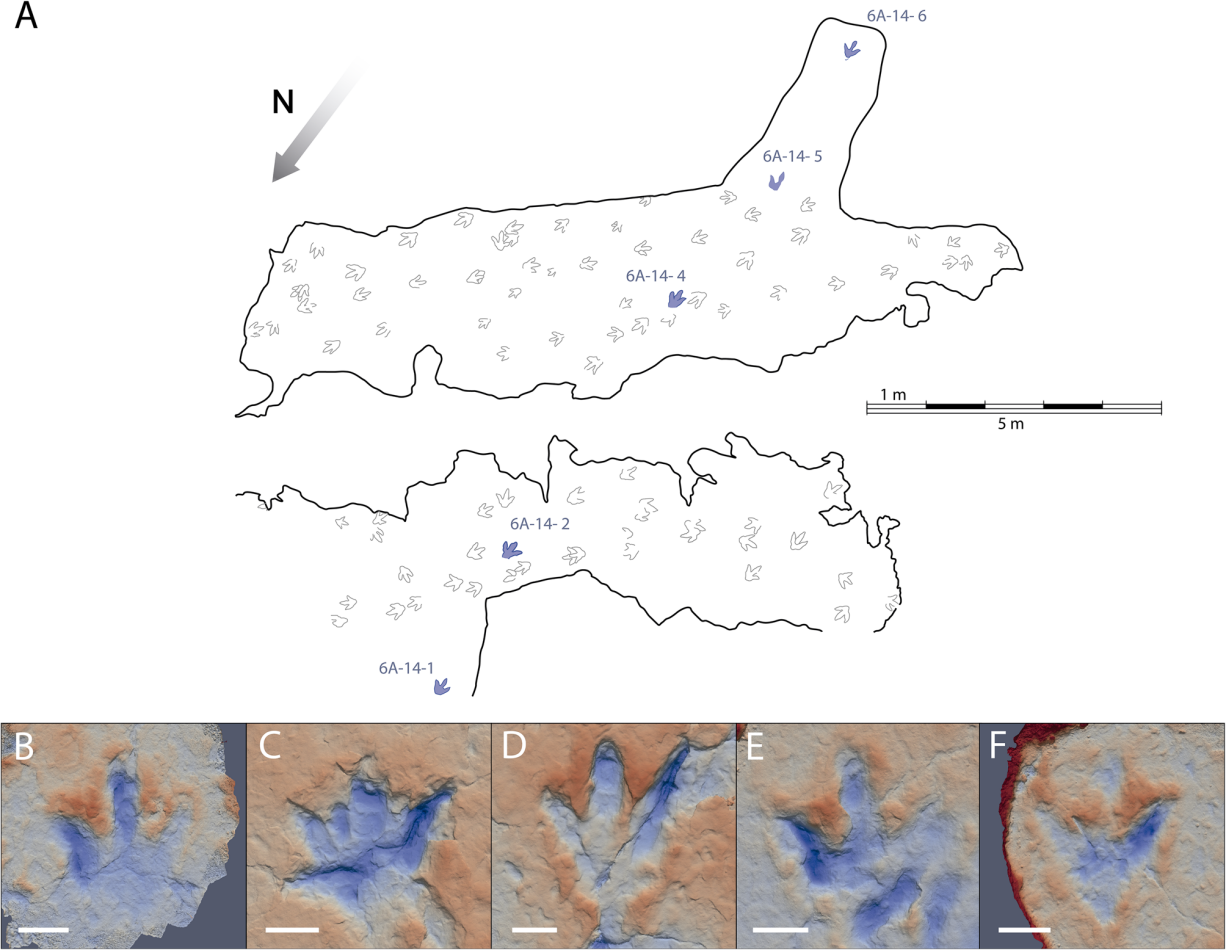
(**A**) La Torre 6A tracksite map with the studied trackway in blue and the other footprints in grey. (**B**–**F**) False-colour maps of the footprints (white scale bar: 10 cm): (**B**) 6A-14-1; (**C**) 6A-14-2; (**D**) 6A-14-4; (**E**) 6A-14-5; (**F**) 6A-14-6.

**Figure 3:**
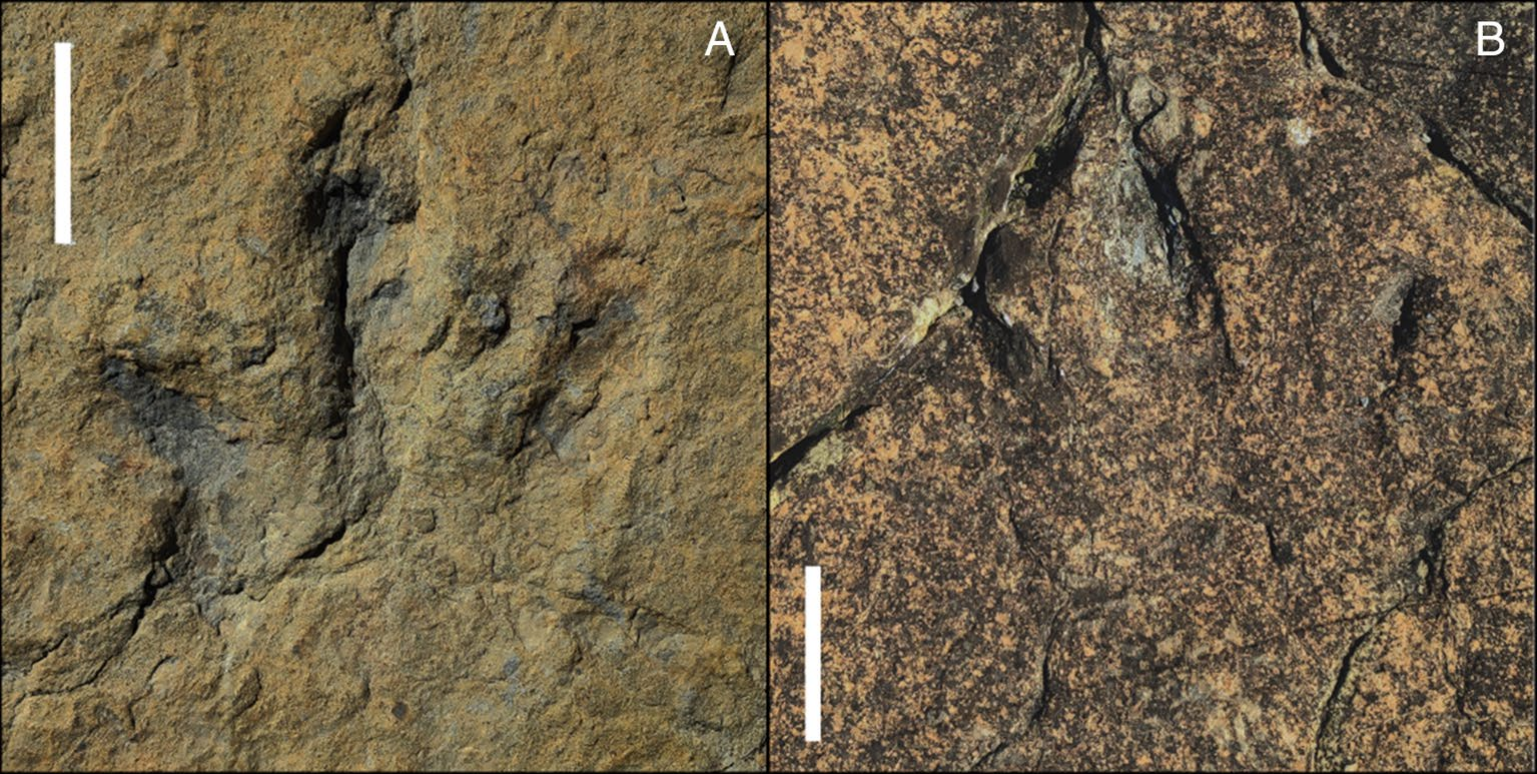
Pictures of (**A**) 6A-14-1 footprint. (**B**) 6B-01-3 footprint. Scale bar = 10 cm.

The La Torre 6A-14 trackway (Fig. 2) preserves only five of the six footprints because the third footprint in the trackway was at a point of the tracksite where the top layers of rock have been lost. The footprints are tridactyl, functionally mesaxonic, and longer than wide (mean length and width, respectively, of 32.8 cm and 30.2 cm). The footprints show well-preserved digit impressions (Fig. 3A). The divarication angle between the digit II and IV impressions is about 67° and varies from 57° to 75°. The metatarsophalangeal area is very shallow in the first footprint and elongated in footprints 2, 4 and 6. The impression of digit II is always deeper than digit IV, and in footprints 2 and 4 a sharp longitudinal groove is preserved, probably related to the claw imprint. The digit III impression also shows a deep area in its distal zone, but the claw imprint is at a higher level than the rest of the digit. In footprint 6, the posterior area of digit III is preserved as a narrow and shallow groove. Pad imprints are identified in footprints 2, 4, and 5. The impression of digit IV is elongated, has a sharp distal end, and is the shallowest of all digits. The mean values for the pace angulation, stride length and pace length are 169°, 523 cm and 265 cm, respectively.

**Figure 4:**
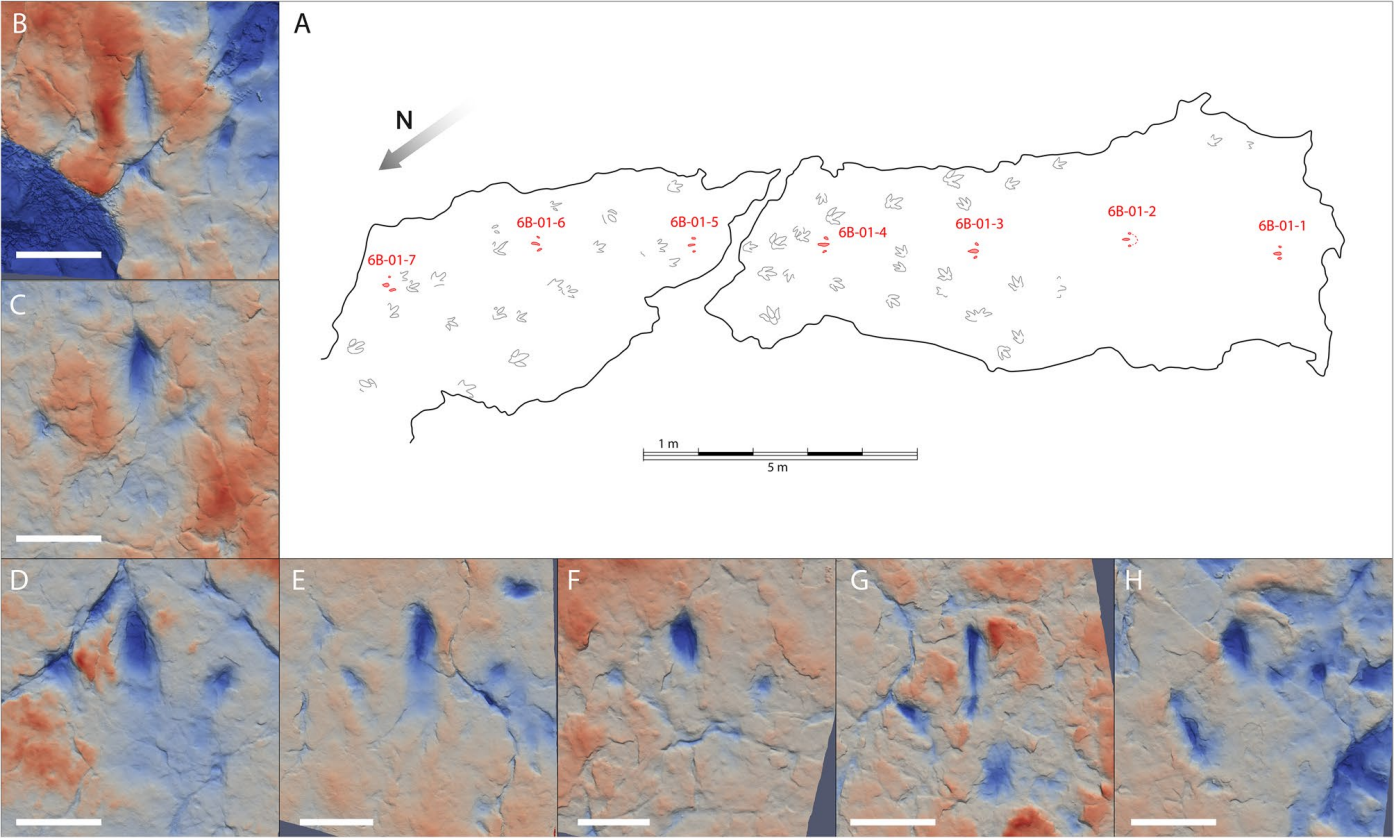
(**A**) La Torre 6B tracksite map with the trackway 6B-01 in red and the other footprints and trackways in grey. (**B**–**H**) False-colour maps of the footprints (white scale bar: 10 cm): (**B**) 6B-01-1; (**C**) 6B-01-2; (**D**) 6B-01-3; (**E**) 6B-01-4; (**F**) 6B-01-5; (**G**) 6B-01-6; (**H**) 6B-01-7.

The La Torre 6B-1trackway (Fig. 4) presents seven footprints. These are tridactyl and functionally mesaxonic. The footprints are longer than wide (mean length and width, respectively, of 28.9 cm and 26.9 cm). The divarication angles of footprints 1 and 2 are 58° and 81°, respectively. The other five footprints have divarication angles ranging from 64° to 74°. While the posterior area is very shallow or not even printed, the digit impressions are deeper and better preserved (Fig. 3B). The digit II impression is mainly characterized by a deep claw trace with a rounded to oval shape and a very shallow posterior part (poorly visible on footprints 1, 3 and 4). The imprint of digit III is elongated, deeper in its anterior area, and presents an acuminate impression that continues as a longitudinal groove along the anterior part of the digit. The digit IV impression is poorly preserved. It is mainly characterized by a discrete oval depression in its anterior part, except for footprint 7, which also preserves the medial surface. The trackway has a mean pace angulation of 172.2°, varying from 164° (angle measured in footprint 6) to 178°. The mean stride and pace lengths are 557.6 cm and 279.6 cm, respectively.

There are other footprints preserved in both tracksites La Torre 6A and 6B with similar features and sizes than those from La Torre 6A-14 and La Torre 6B-1^24^. They are tridactyl, functionally mesaxonic and longer than wide, with lengths around 30 cm. The preservation is also similar, with digits generally better printed than the metatarsophalangeal area. The trackways present pace and stride lengths shorter than La Torre 6A-14 and 6B-1 trackway with strides between 67 and 122 cm^24^.

### Speed analysis

**Figure 5:**
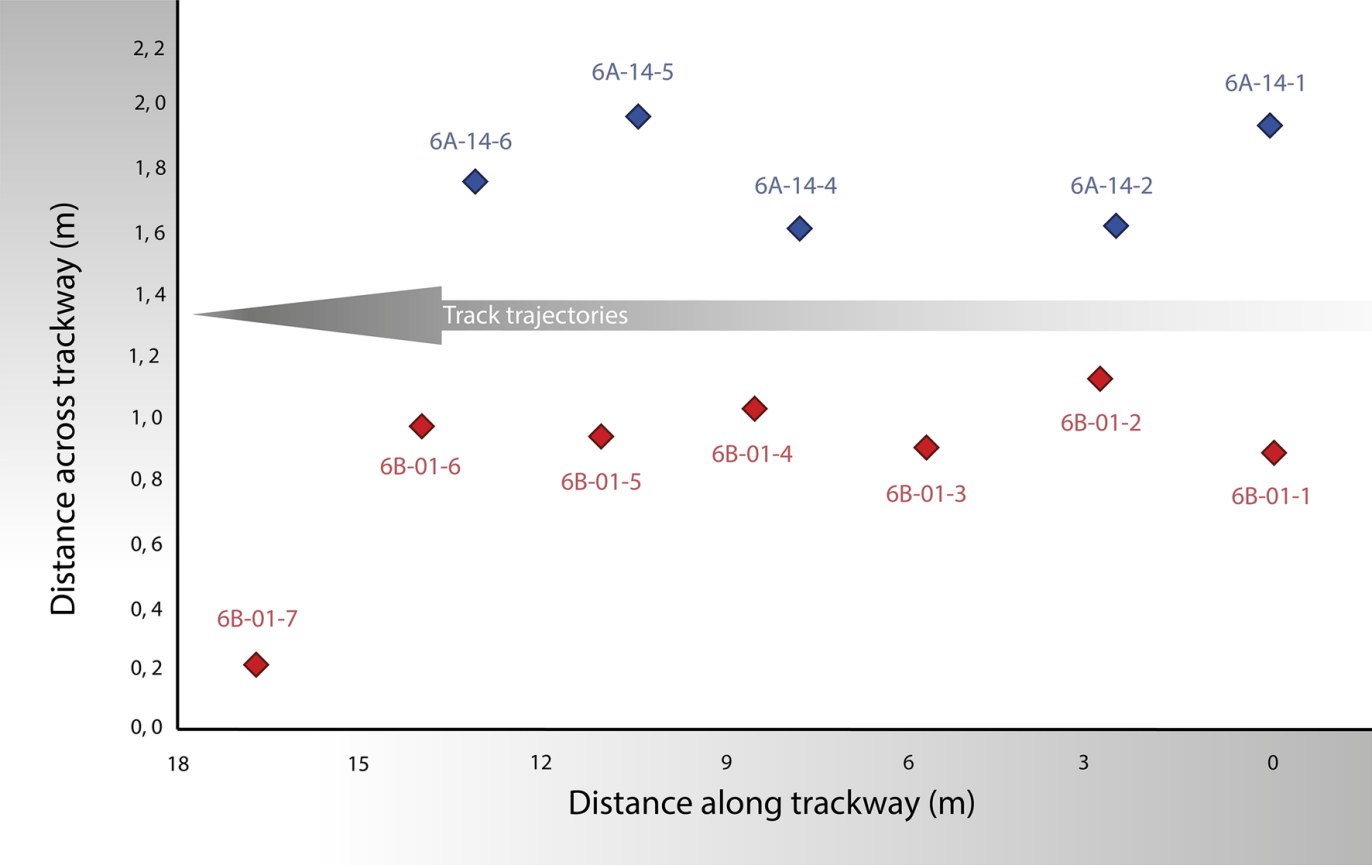
Positions of the measurement points (marked by the anterior tip of the central digit) for the tracks of La Torre 6A (blue, top) and 6B (red, bottom). To fit the trajectory of the La Torre 6A trackway, all the recovered footprints are used, but for the La Torre 6B trackway only the first six footprints are used, because the seventh footprint of this trackway is clearly shifted leftward. The length of the entire La Torre 6A trackway is 13.07 m. The origin of the distances along the tracks is located at the first measurement point on the first footprint of each trackway. The origin of the distances across the tracks is arbitrarily placed.

Table 1 and Fig. 5 show the reference *x* and *y* positions of individual footprints along both trackways, with measurements from the digital 3D models for each track, based on the orthomosaics obtained from the 3D models. For fitting purposes, for the La Torre 6A trackway all the recovered footprints were used, whereas for the La Torre 6B trackway only the positions of the first six footprints were used because the seventh footprint is clearly shifted leftward, indicating a certain change in the displacement direction (see Figs. 4 and 5). The length of the entire La Torre 6A trackway is 13.07 m, whereas the length of the first six footprints of La Torre 6B is 13.93 m. Because these lengths correspond to 2.5 strides, the mean stride lengths are, respectively, 523 cm and 557 cm for the La Torre 6A-14 and 6B-1 trackways.

**Table 1:** Footprint lengths and positions (given by the anterior tip of the central digit) for La Torre 6A and 6B trackways.

Footprint number	Footprint length (cm)	Footprint positions^a,b^ *x*(m); *y*(m)
**La Torre 6A trackway footprints**		
6A-14-1	35.2	0.000; 0.328
6A-14-2	32.3	2.530; 0.004
6A-14-3	–	–
6A-14-4	35.7	7.764; 0.000
6A-14-5	28.5?	10.408; 0.355
6A-14-6	30.0	13.067; 0.147
**La Torre 6B trackway footprints**		
6B-01-1	29.8	0.000; 0.000
6B-01-2	24.9?	2.783; 0.242
6B-01-3	27.1	5.616; 0.020
6B-01-4	32.0?	8.414; 0.148
6B-01-5	27.8?	11.062; 0.057
6B-01-6	29.9	13.929; 0.086
6B-01-7	28.3?	16.659; − 0.672

See main text for further explanations on the orientation of measurement points.

^a^The origin of distances along the *x* axis (following track direction) is located at the measurement point on the first footprint of each trackway.

^b^The origin of distances along the *y* axis (perpendicular to track trajectory) is located on the leftward footprint with respect to the trajectory direction; in the case of the La Torre 6B trackway, footprint 6B-01-7 was not included in the track-trajectory fitting, and for this reason its *y* position has a negative value.

In calculating footprint lengths, only those for which reliable measurements can be made are considered (see Table 1). The result is 32.8 ± 3.1 cm (n = 4) and 28.9 ± 1.3 cm (n = 3), respectively, for the La Torre 6A and 6B trackways. This translates into hip heights of 1.19–1.44 and 1.10–1.21 m for the theropods printing the respective tracks. The *λ/h* ratios for both trackways (Table 2) are between 3.5 and 5.0, much higher than those considered to indicate the start of running, which are usually around two for bipedal animals^9,29^.

**Table 2:** Results of the speed analysis for La Torre 6A and 6B trackways.

	La Torre 6A	La Torre 6B
Footprint length (cm)^a^	32.8 ± 3.1	28.9 ± 1.3
Average stride length (m)	5.23	5.57
λ/*h* ratio^b^	3.6–4.4	4.6–5.0
Mean speed (m s^−1^)	6.5–10.3 [8.2–9.0]	8.8–12.4 [10.5–11.6]
Mean speed (km h^−1^)	23.3–36.9 [29.4–32.5]	31.6–44.7 [37.8–41.9]

The footprint and mean stride lengths used in the calculations are indicated. The full ranges of speeds take into account uncertainties in footprint length (and hence in hip height) and the ± 12% uncertainty associated with Eq. (1); the lower and higher values (in the bracketed ranges) were calculated, respectively, from Eq. (1) and Alexander’s equation.

^a^Calculated from footprint measurements without question marks in Table 1.

^b^*h* is equal to 4 times the footprint length; the λ/*h* ratio is given as a range.

**Figure 6:**
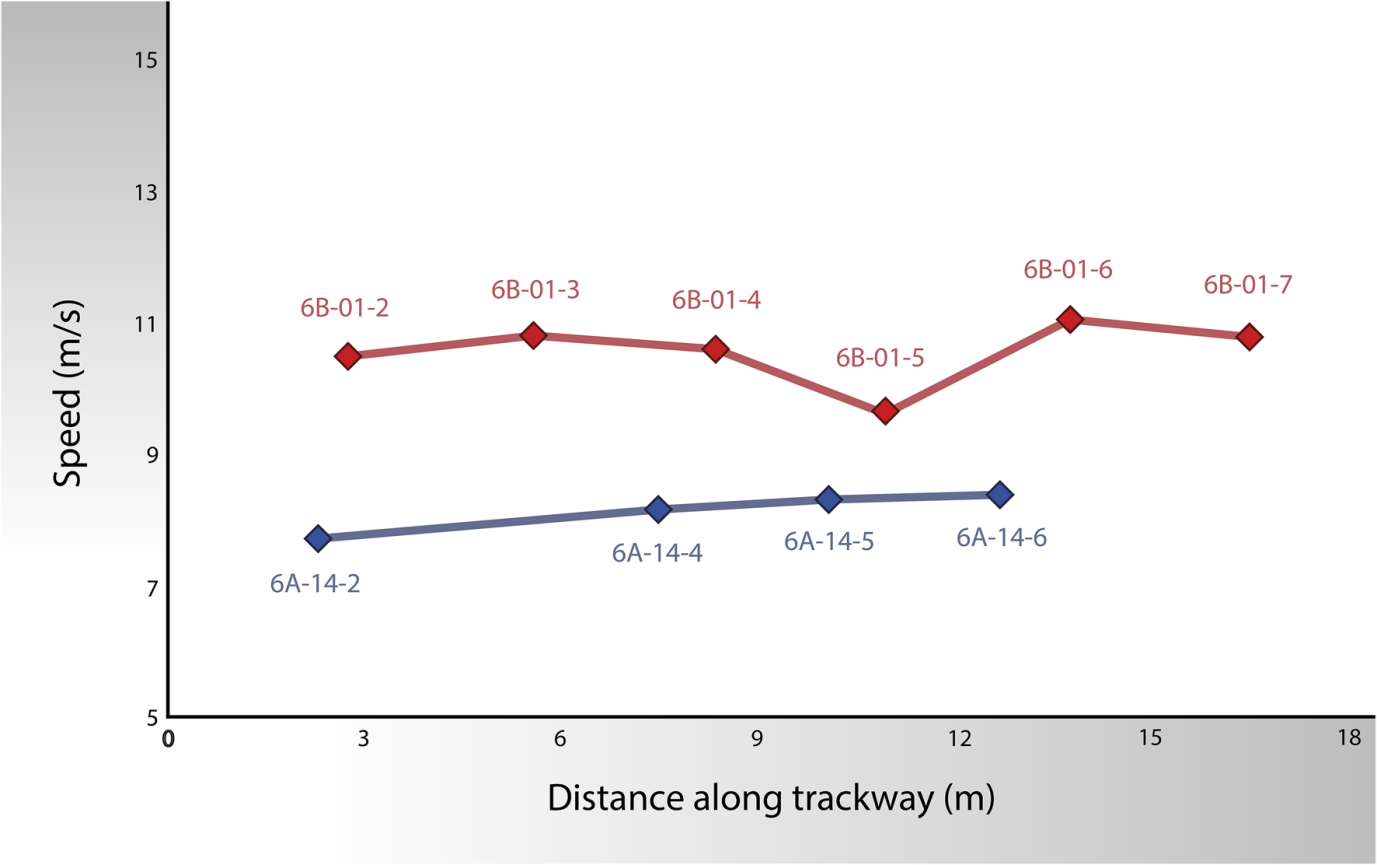
Step-by-step speed changes calculated for the tracks of La Torre 6A (blue, top) and 6B (red, bottom). Speeds are marked at the positions of the measurement point of the final footprint of each individual step. Only speeds calculated for the mean footprint lengths and central results given by Eq. (1) are represented, in order to avoid propagating uncertainties and to focus on identifying the occurrence, or not, of speed changes along the tracks. Footprint 6A-14-3 is not preserved, and in this case the speed is calculated for the stride between footprints 6A-14-2 and 6A-14-4. Due to the rectified trajectory, the speed between footprints 6B-01-6 and 6B-01-7 is calculated for a step of 2.87 m, corresponding to the linear distance between the footprints (and therefore for the pace length, not for the difference in *x* position given in Table 1).

**Figure 7:**
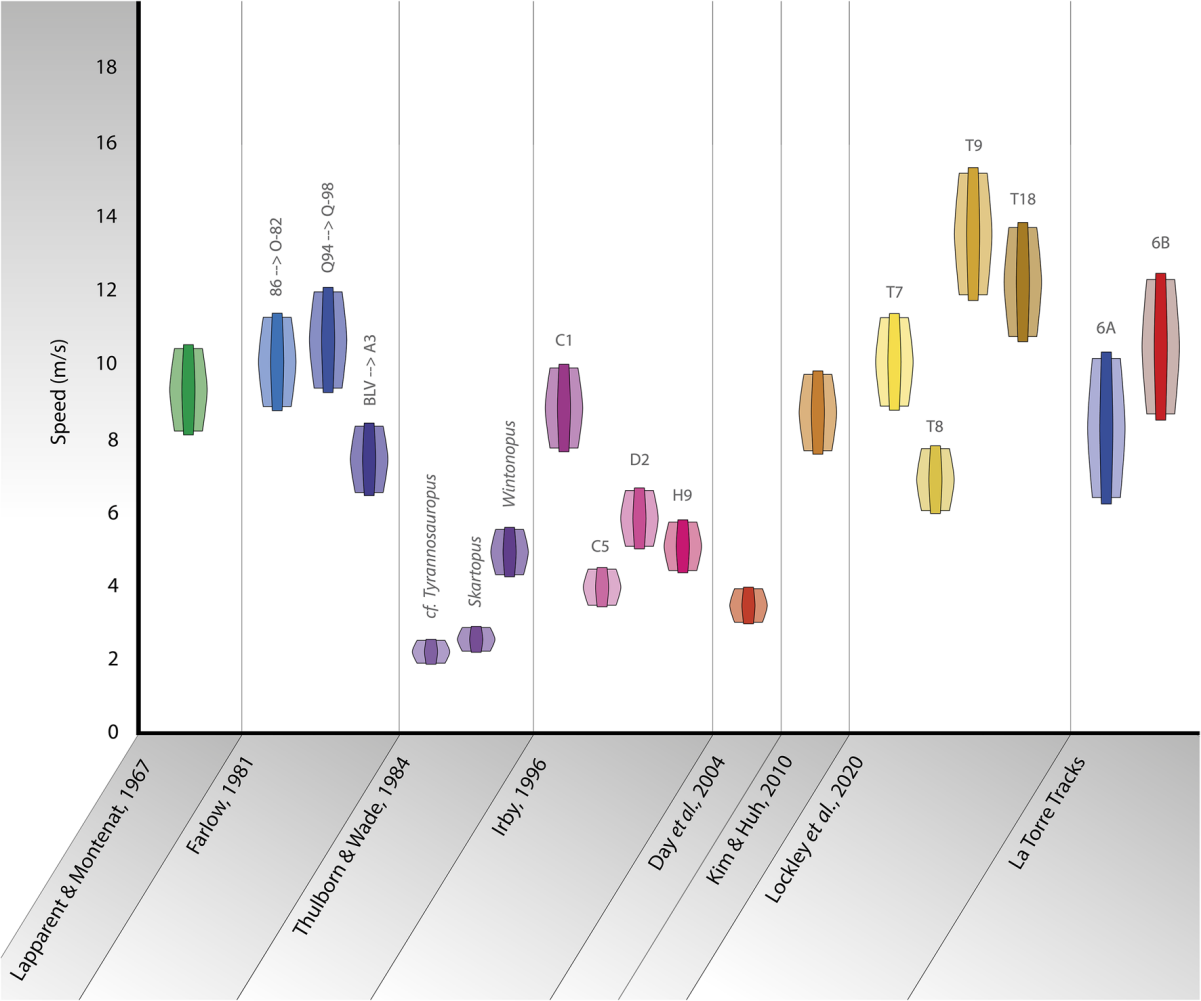
Comparison of the top fastest dinosaur runners identified by tracks, including the dinosaurs from La Torre 6A and 6B.

Table 2 and Fig. 6 present the results of the speed analysis obtained in this study. All the speed ranges take account of the uncertainty in footprint lengths, as well as the ± 12% uncertainty associated with Eq. (1); we also indicate the results given by Eq. (1) and by Alexander’s equation (which does not quote an associated uncertainty). The speeds obtained for both trackways are high and again indicate running animals (Table 2). Based on Eq. (1), the speed obtained for the La Torre 6A theropod is between 6.5 and 10.3 ms^−1^, whereas the La Torre 6B theropod ran even faster at speeds of between 8.8 and 12.4 ms^−1^. These speeds are among the fastest calculated for dinosaurs from fossil tracks^17,23^ (see Table 3 and Fig. 7). In fact, La Torre 6B records (to our knowledge) one of the fastest trackmaker dinosaurs currently known. The two dinosaurs believed to be faster were reported by Lockley et al.^23^ from the Early Jurassic of San Juan County (Utah) and by Farlow^17^ from the Early Cretaceous of F6 Ranch (Texas), for which these authors calculated speeds of 13.7 and 11.8 ms^−1^, respectively, following Alexander’s method^9^. Based on stride and footprint lengths from these authors, Eq. (1) predicts speeds of 10.8–13.8 and 9.4–11.9 ms^−1^ for the trackways from Utah and Texas, respectively; see our results in Table 2 for comparison. Interestingly, Lockley et al.^23^ noted that the fastest speeds calculated from the tracks of Utah and Texas correspond to footprint lengths in the range between 29 and 39 cm. This is also true for both tracks from La Torre.

**Table 3:** (a) Theoretical maximum speeds obtained from physical dynamic approaches and (b) speeds calculated from different tracks and studies.

(a)			
Source	Taxon/track	Max. speed (ms^−1^)	
Hutchinson^30^	*Tyrannosaurus rex*	< 11	
	*Tyrannosaurus rex* (young)	11 to 14	
	*Coelophysis*	9	
Sellers and Manning^6^	*Compsognathus*	17.8	
	*Velociraptor*	10.8	
	*Dilophosaurus*	10.5	
	*Allosaurus*	9.4	
	*Tyrannosaurus*	8	
Hirt et al.^31^	*Velociraptor*	15.15	
	*Allosaurus*	11.32	
	*Tyrannosaurus*	7.51	

(b)			
Source	Taxon/track	v^29^	v^9^
Kim and Huh^22^	Trackway B	7.51–9.55	9.43
Lapparent and Montenat^16^	*Saltopoides igalensis*	8.26–10.54	10.38
Thulborn and Wade^18^	cf. *Tyrannosauropus*	1.99–2.54	2.51
	*Skartopus*	2.39–3.04	3.01
	*Wintonopus*	4.39–5.59	5.52
Farlow^17^	86-0-82 Mean	8.89–11.31	11.17
	Q94-Q98 Mean	9.45–12.03	11.88
	BLV-A3 Mean	6.55–8.33	8.23
Irby^20^	C1	7.80–9.93	9.8
	C5	3.53–4.50	4.44
	D2	5.21–6.64	6.56
	H9	4.65–5.92	5.85
Day et al.^32^	T13	3.26–4.16	4.11
Lockley et al.^23^	T7	8.99–11.44	11.3
	T8	6.15–7.82	7.73
	T9^a^	12.05–15.33	15.14
	T18	10.89–13.86	13.69
La Torre tracks	6A-14	6.5–10.3	8.2–9.0
	6B-01	8.8–12.4	10.5–11.6

Equation (1) was used to recalculate all speeds with the data given in each publication applying in each case the relation *h* = 4*FL*. In the case of tracks with a high variation in SL (Stride Length), Eq. (1) was applied to the longest stride with a mean FL.

^a^The trackway is composed of just two footprints.

Furthermore, step-by-step speeds are interesting for shedding light on speed changes and the possible behaviour recorded in the trackways^13,22,33^. To calculate step-by-step speeds, we consider the length of each individual step, measured in the direction of the trackways (i.e., the difference in *x* positions of two consecutive footprints). In this case we use *λ* = 2*S* in Eq. (1), where *S* is the length of an individual step. Our results are shown in Fig. 7. We only represent the cases for the mean footprint lengths, and central results given by Eq. (1), to focus on what is relevant here: identifying the occurrence, or not, of changes in speed along the trackways. In the case of the La Torre 6A trackway the speed increases smoothly along the recovered track. In the case of the La Torre 6B trackway, there is a substantial speed reduction between footprints 6B-01-4 and 6B-01-5, and again a substantial increase between 6B-01-5 and 6B-01-6. There is a new reduction between 6B-01-6 and 6B-01-7, but in this case there is also a change in direction. It is not possible to be sure whether the two changes were related, but it is an interesting possibility.

### Possible trackmakers

Due to the conjunction of features present in the footprints of the La Torre 6A and 6B running trackways (e.g., claw imprints in some footprints, narrow and elongated digit impressions, high pace angles), this study concludes that the trackmakers were theropods. The two trackways present several similarities, such as the L/W ratio, the pace angulation, and digit impressions deeper than the posterior area. Indeed, the best-preserved footprints of both trackways, 6A-14-1 and 6B-01-3, are very similar in shape. Nevertheless, they also show some differences. The footprints of La Torre 6B preserve a very shallow metatarsophalangeal area, whereas in La Torre 6A some of the footprints show a long metatarsal impression. Thus, the trackmakers of both trackways probably belong to the same taxonomic group, the differences between the trackways being a product of variations in the consistency of the substrate and/or in the locomotion pattern. The idea that the same individual could have generated both tracks can be ruled out due to the mean values for length and width, which show the footprints of the La Torre 6A trackway to be bigger than those of the La Torre 6B trackway. Actualist investigations into hominid tracks have suggested an intraindividual dispersion of 12.8% in size along the same trackway^34,35^. In dinosaurs, differences of more than 20% in the lengths of footprints along the same trackway are also reported^36^. But the tracks from La Torre 6A and 6B are each uniform in their lengths and widths and seem to be uniformly different from one another in their sizes, the trackmaker from La Torre 6A being bigger than that from La Torre 6B.

Identifying the trackmakers as belonging to a particular theropod group or genus is not possible, but the size, proportions and features of the footprints, the pace angles, and the speeds calculated indicate that the trackmaker was a very agile, medium-sized, non-avian theropod. Medium-sized theropods from the Early Cretaceous of the Iberian Peninsula include the following three groups. (1) Spinosaurids have been identified in the Early Cretaceous of Iberia on the basis of isolated teeth^28,37–41^ and skeletal remains^42–45^, *Vallibonavenatrix cani* being the only spinosaurid genus and species described to date^46^. (2) Carcharodontosaurids are known in several Early Cretaceous localities by teeth^40^ and skeletal remains^47^; the only genus and species currently described is the iconic *Concavenator corcovatus*^48,49^. Moratalla et al.^50^ published a trackway from Las Hoyas (Cuenca, Spain) and suggested its possible trackmaker to be *Concavenator*. The footprints from Las Hoyas differ in some features from the footprints from both La Torre 6A and 6B (divarication angle, pace angle, footprint outline), but are similar in size and proportions. If the hip height/footprint length ratio of 4 is applied, the hip height of the Las Hoyas trackmaker proves to be 104–112 cm, which is a similar value to that calculated for the tracks of La Torre 6A (119–144 cm) and La Torre 6B (110–121 cm). Finally 3) ceratosaurian theropods have been included as components of Early Cretaceous Iberian theropod biodiversity, but *Camarillasaurus cirugedae* is currently regarded as a spinosaurid and no longer as a ceratosaur^51,52^. In addition to *Camarillasaurus*, the presence of ceratosaurians in the Late Jurassic of Portugal has been suggested on the basis of isolated teeth^53,54^ and dental remains of cf. Abelisauridae have been indentified in the Cenomanian of Algora^55^; and the presence of *Genusaurus sisteronis* has been established in the Albian of Provence in France^56^.

### Fastest dinosaurs

One of the most intriguing and key features of non-avian dinosaurs in terms of their behaviour and capacities is the speed and kind of movement that they were able to perform^1,2,57^. The form of bipedalism present in some dinosaur groups, especially in theropods and ornithopods, is not present in any extant animal, complicating the comparison of results. Birds share many of the key features observed in non-avian bipedal dinosaurs, but the reduction and loss of the tail and the modification of posture during evolution have changed their mode of movement over the course of time^58–60^.

Many works have tried to shed light on dinosaur locomotion in terms of the speed and kind of movement, through two major approaches: (1) biomechanical models based on musculoskeletal reconstructions and the application of physical dynamics to these; following this approach, many works have proposed running abilities and maximum speeds attainable by non-avian dinosaurs^6,30,31,61–63^; (2) speed estimates based on physical kinematics, linking stride length and speed with their tracks^9–11,64–67^.

Physical dynamic models for bipedal dinosaurs propose that there is a major change in running abilities when size becomes important^57^, specifically in the range of 100–1000 kg^61^. When approaching masses greater than a tonne, bipedal non-avian dinosaurs would display lower running abilities due to the higher muscular masses needed to support the forces and stresses derived from high velocities^61^. Furthermore, larger animals achieve lower acceleration due to their progressively bigger mass in relation to muscular performance, leading to a depletion of readily mobilizable energy before reaching theoretically maximum speeds^63^. Table 3 shows several theoretical top speeds obtained from physical dynamic models and speed estimates calculated from fossil tracks of running dinosaurs; the speeds were recalculated from Eq. (1) and taking *h*/(footprint length) = 4, in order to draw direct comparisons between tracks published by different authors (although we also indicate the speed given by Alexander’s^9^ equation); results are shown in Table 3b and Fig. 7.

The size of the La Torre 6A and 6B footprints are in the range of theoretical “good runner” dinosaurs proposed by ichnological data and biomechanical models. Ichnological data suggest that the fastest non-avian dinosaur speeds are found in tracks with footprints between 29 and 39 cm long^14,23^. This fits with the theoretical estimation of maximum speeds obtained with biomechanical models based on musculoskeletal systems, which propose that non-avian dinosaurs in the range of 100–1000 kg were still fast dinosaurs able to reach high top speeds^7,61,63^. This could be partially explained by the great selection pressure for higher top speeds in dinosaurs with masses inferior to 1000 kg, because of their double condition as the hunters of smaller prey and the prey of bigger hunters^7^.

The trackway from La Torre 6A shows a smooth and constant increase in the estimated speed along the track. Changes in speed are scarce in the ichnofossil record, but there are some clear examples. One of the clearest changes in speed published is the case studied by Kim and Huh^22^, where a clear acceleration phase was recorded, similar to that shown by Weems^13^. These changes in speed occur rapidly, with a significant increase in speed in a short time span, comprising 3–4 steps. However, the case described by Kim and Huh^22^ is remarkable, for previous footprints show a smooth and continuous increase in speed, similar to that seen in the La Torre 6A trackway. This shows that dinosaurs were able to increase their speed in two different ways, either an abrupt increase in their displacement speed or a smooth and constant acceleration, and that they were able to combine both strategies within a single run phase. By contrast with La Torre 6A, the La Torre 6B trackway shows significant abrupt (from one step to the next) speed changes, again suggesting a “manoeuvring” dinosaur.

The speeds calculated for both trackways from La Torre are among the top three speeds ever calculated for non-avian theropod tracks. Moreover, the La Torre 6B trackway at least was printed by a dinosaur with the ability to make and control substantial speed changes while running. The La Torre 6A-14 and La Torre 6B-1 trackways studied in the present paper share with other ichnofossil localities (see Table 3) a record of two or more running theropods. Thus, it seems that some ecological conditions were conducive to medium-sized theropods moving by running.

## Materials and methods

### Geographical and geological context

The La Torre 6A and La Torre 6B tracksites crop out in the locality of Igea, situated in the Comarca of Cervera (southeast La Rioja, Spain), and they are located on the northern limb of the Cerro Mountain, called Umbría de La Torre, northwest of the town of Igea.

Geologically, the tracksites are located in the northeastern sector of Cameros Basin (Fig. 1). This basin was formed in the second rifting stage that occurred during the Late Jurassic and Early Cretaceous, along with the other sedimentary basins that constitute the Iberian Mesozoic Rift^68^. The basin has been traditionally divided into two different sectors, showing important differences in their stratigraphy and evolution.

- The northeastern sector (East Cameros) of the basin presents more than 5000 m in thickness due to the high rates of subsidence^69,70^ and is dated as Tithonian to early Albian^71^.
- The southwestern sector (West Cameros) shows a more modest subsidence rate with sediments reaching up to 2500 m in thickness dated as Kimmeridgian to Early Albian^72,73^.

The synrift deposits of East Cameros have been traditionally divided into five groups called Tera, Oncala, Urbión, Enciso and Oliván^74^ or into eight depositional sequences (DS1-DS8), as proposed by Mas et al.^68^.

La Torre 6A and 6B crop out in the Enciso Group or DS7 according to Mas et al.^68^. DS7 is more than 2000 m in thickness^75^ and is composed of the Jubera Formation, the Leza Formation, and the carbonate-siliciclastic deposits of the Enciso Group^76^. In the main depocentre of the Cameros Basin, the Enciso Group is represented by mixed carbonate-siliciclastic deposits^76^, interpreted as a siliciclastic-influenced lacustrine and palustrine environment^68–70^. Northwards, these deposits overlie the Leza Formation^76^. Furthermore, the Leza and Jubera formations are genetically related^76,77^, being formed in a coastal-wetland environment and alluvial fans, respectively^70,76,77^.

The La Torre 6A and 6B tracksites are situated in the upper part of the Enciso Group in mixed carbonate-siliciclastic deposits. Stratigraphically, the studied area is composed of an alternation of marls and limestones with signs of subaerial exposure, such as dinosaur tracks and mud cracks. These facies have been interpreted as palustrine periods in which the water level fell for a short timespan associated with two similar contexts^78^: (1) palustrine deposits formed in the intertidal areas of an important lacustrine system; or (2) deposits of a small carbonate lake, developed in avulsive areas, probably related to lacustrine deltaic dynamics.

Despite the abundance of levels with footprints, only the beds of La Torre 6A and 6B have a surface exposed enough to characterize and study the tracks. Both tracksites are preserved at the top of the same track-bearing surface; they are 30 m apart, the area between them covered by Quaternary deposits and vegetation. In their surface, moreover, the tracksites show accumulations of vegetal/algal remains and ostracods, interpreted as transported either by the wind (vegetal remains) or by water when the lake level was low (ostracods and algae)^78^. A thin layer of marls crops out above the tracksite level, indicating a low-energy environment that could have protected the tracksite level with sedimentation of siliciclastic and carbonate material.

As regards the age of the Enciso Group, DS7 has been dated as late Barremian-early Aptian based on biostratigraphic and sedimentological data, e.g.^69,76,77,79^. Nevertheless, Hernán^78^ has proposed a temporal range of 5.57 Ma for the Enciso Group and assigned a late Barremian-late Aptian age to the Enciso Group.

### Tracksite

La Torre is a set of 14 tracksites (La Torre 1A, 1B, 2, 3, 3A, 3B, 3C, 4, 5, 5A, 6A, 6B, 6C and L) initially studied by Agirrezabala et al.^24^ close to the village of Igea (La Rioja province, Spain). Specifically, these authors identified 14 trackways and 15 isolated footprints (92 footprints in total) in La Torre 6A, and 34 trackways and 47 isolated footprints (145 footprints in total) in La Torre 6B. Among all these trackways, two of them stand out as possible evidence of running non-avian theropods, the La Torre 6A-14 and La Torre 6B-1 trackways. The trackway from La Torre 6A has six footprints. It is composed of two of the isolated footprints studied by Agirrezabala et al.^24^, three newly excavated footprints, and one, the third in the trackway, which is eroded. The La Torre 6B-1 trackway preserves seven footprints, six of them belong to trackway 1 of Agirrezabala et al.^24^ and a newly excavated footprint (the seventh).

The studied footprints are preserved in situ as concave epireliefs at the top of the same limestone level. They are covered by non-deformed laminated marls. There are no thin layers inside the footprints, so the presence of overtracks or underprints (sensu Marty^80^; Marty et al.^81^) is ruled out. Thus, the studied surface (sensu Requeta et al.^82^) is the tracking surface^83^, and the footprints are true tracks (sensu Lockley^84^).

### Model generation and measurements

Measurements were taken both in situ and from photogrammetric 3D models. The models were generated from pictures taken by a Canon EOS 1200D with an EF-S 18–55 mm II lens using Agisoft Metashape Professional (v 1.6.1). Two different methods were used in generating the model: (1) modelling the trackway, and (2) modelling individual footprints. For trackway modelling, zenithal photographs were taken to generate an orthomosaic that would allow measurements to be made all along the trackway. To model La Torre 6B, 200 zenithal photos were taken to generate the orthomosaic and the 3D model; for the La Torre 6A trackway, 176 photos were taken to generate the orthomosaic and the general 3D model. For individual footprint modelling, several photographs were taken from different views and orientations to generate a precise three-dimensional model of each footprint that allowed us not just to make measurements, but also to observe and analyse their shape and details. In modelling each footprint, between 50 and 60 photos were taken to obtain a high-resolution model that would reflect small details and features, with an element size of 1.5 mm in the areas with a more complex geometry. Once obtained and scaled, the meshes were exported as .stl files and imported into ParaView (v 5.9.0-RC2), where false-colour depth maps and measurements were made. In addition to 3D models, orthomosaics were obtained too, in order to provide the *x* and *y* coordinates of the tracksites, establishing the mid-point of the models as (0, 0).

### Speed analysis

The speeds for both trackmakers were calculated based on the concept of dynamic similarity, which states that living and extinct animals share common basic mechanical properties^9^. We use the relation between stride length and speed following the updated equation^29^

1 $${\text{v}}\left( { \pm {12}\% } \right) = 0.{226}\,g^{{0.{5}}} \,\lambda^{{{1}.{67}}} \, h^{{ - {1}.{17}}} ,$$

where *v* is speed, *g* (= 9.8 ms^−1^) is the acceleration due to gravity, *λ* is the absolute stride length (defined as the distance between the equivalent points of two consecutive footprints generated by the same foot), and *h* is the total hip height. This equation was chosen because Ruiz and Torices^29^ based their conclusions on an expanded dataset for humans walking and running and discovered a potential relation of *λ*^5/3^ (identical to that deduced by Alexander^9^). Equation (1) differs from Alexander’s relation only in a proportionality constant of 2.26 instead 2.5, works well for bipedally running humans, and quotes an uncertainty range that includes the results obtained with Alexander’s equation. The equation of Thulborn and Wade^18^ for running dinosaurs was not used, because this is based on the relation found by Alexander et al.^64^ for quadrupedally running ungulates, which are not the best equivalent for bipedal animals.

Mean speeds, as well as step-by-step speeds, were calculated along the recovered trackways. Stride and step lengths were measured taking the anterior tip of the print of the central digit as a reference point, because this point is easy to locate in footprints that are not well printed or preserved and allows a consistent systematization for the measurements. From the positions of the individual footprints obtained in this way, the mean direction of each track was worked out through a least-squares linear fit. Step lengths were then taken as the distances, along the deduced mean direction of the track, between the equivalent points of two consecutive footprints generated by different feet.

To calculate the height to the hip articulation *h*, the standard *h*/ (footprint length) ratio of 4 proposed by Alexander^9^ and Henderson^85^ was used. Although several authors have preferred to give a range for the hip height/footprint length ratio depending on the kind of animal, e.g.^11,18^. The use of a *h*/(footprint length) ratio of 4 is useful for two reasons: (1) it is close to the upper bound of the ranges obtained for theropods (2.8–4.2) according to the reassessment by Rainforth and Manzella^86^ and therefore gives relatively conservative speed estimates; (2) it permits good comparisons with most speed calculations for running dinosaurs, which have used the same value for the *h*/(footprint length) ratio.
